# The contribution of neutrophils to bacteriophage clearance and pharmacokinetics in vivo

**DOI:** 10.1172/jci.insight.181309

**Published:** 2024-10-22

**Authors:** Arne Echterhof, Tejas Dharmaraj, Arya Khosravi, Robert McBride, Lynn Miesel, Ju-Hsin Chia, Patrick M. Blankenberg, Kun-Yuan Lin, Chien-Chang Shen, Yu-Ling Lee, Yu-Chuan Yeh, Wei Ting Liao, Francis G. Blankenberg, Krystyna Dąbrowska, Derek F. Amanatullah, Adam R. Frymoyer, Paul L. Bollyky

**Affiliations:** 1 Division of Infectious Diseases and Geographic Medicine, Department of Medicine, Stanford University School of Medicine, Stanford, California, USA.; 2 Institute of Medical Microbiology, University Hospital of Muenster, Muenster, Germany.; 3 Felix Biotechnology, South San Francisco, California, USA.; 4 Pharmacology Discovery Services, Taipei, Taiwan.; 5 Division of Pediatric Radiology and Nuclear Medicine, Department of Radiology, Lucile Packard Children’s Hospital, Stanford, California, USA.; 6 Laboratory of Phage Molecular Biology, Institute of Immunology and Experimental Therapy, Polish Academy of Sciences, Wrocław, Poland.; 7 Department of Orthopedic Surgery, Stanford University School of Medicine, Stanford, California, USA.; 8 Division of Neonatal and Developmental Medicine, Department of Pediatrics, Stanford University School of Medicine, Palo Alto, California, USA.

**Keywords:** Infectious disease, Bacterial infections

## Abstract

With the increasing prevalence of antimicrobial-resistant bacterial infections, there is interest in using bacteriophages (phages) to treat such infections. However, the factors that govern bacteriophage pharmacokinetics in vivo remain poorly understood. Here, we have examined the contribution of neutrophils, the most abundant phagocytes in the body, to the pharmacokinetics of i.v. administered bacteriophage in uninfected mice. A single dose of LPS-5, a bacteriophage recently used in human clinical trials to treat drug-resistant *Pseudomonas aeruginosa*, was administered i.v. to both immunocompetent BALB/c and neutropenic CD1 mice. Phage concentrations were assessed in peripheral blood and spleen at 0.25, 1, 2, 4, 8, 12, and 24 hours after administration by plaque assay and qPCR. We observed that the phage clearance was only minimally affected by neutropenia. Indeed, the half-lives of phages in blood in BALB/c and CD1 mice were 3.45 and 3.66 hours, respectively. These data suggest that neutrophil-mediated phagocytosis is not a major determinant of phage clearance. Conversely, we observed a substantial discrepancy in circulating phage levels over time when measured by qPCR versus plaque assay, suggesting that significant inactivation of circulating phages occurs over time. These data indicate that alternative factors, but not neutrophils, inactivate i.v. administered phages.

## Abstract 2

Bacteriophage therapy is a promising solution for treatment of chronic bacterial infections. Here, we have investigated the role of neutrophils in bacteriophage clearance in mice.

## Introduction

The global crisis of bacterial antimicrobial resistance (AMR) represents a profound challenge to human health. Once-treatable infections are now responsible for extensive morbidity and mortality and have the potential to evolve into global health problems of pandemic dimensions (1).

*Pseudomonas aeruginosa* is one of the most problematic pathogens for which new treatment options are needed (2–4). Multidrug-resistant (MDR) strains of *P*. *aeruginosa* are prevalent in environmental and clinical settings due to acquired resistance and intrinsic mechanisms of resistance (5, 6). There is a need for innovative treatments to treat MDR *P*. *aeruginosa* and other pathogens.

Bacteriophages (phages), viruses that kill bacteria, are an exciting treatment option for AMR bacterial infections (7–10). Multiple studies have demonstrated the efficacy of phage therapy against MDR *P*. *aeruginosa* (11–14), either alone or in conjunction with conventional antibiotics (15). Phage therapy is saving lives; however, success has been inconsistent (15–18). This has limited the therapeutic and commercial prospects of this approach.

Critical to the successful development of phages as a therapeutic treatment will be a deep understanding of its pharmacokinetics (PK) (19). While there is, in fact, a large body of literature on phage therapy, it is heterogenous; difficult to access, as much of it is in the non-English-language literature; and often predates the current era of phage therapy. Fortunately, this literature has been summarized in a recent review (20).

A common method of delivering therapeutic phages is intravenous (i.v.) administration, particularly for systemic and pulmonary infections (21). After i.v. administration, the half-life (*t*_1/2_) of phages in the blood of mice has been reported as between 2.2 hours and 4.5 hours in different animal models (22, 23). Most phages are cleared from the blood by the liver and spleen within minutes to hours (24–26), and i.v. administration of phages leads to phage accumulation in these tissues. The rapid removal of phages from circulation and subsequent degradation limits their potential therapeutic efficacy (27).

Endocytosis plays a prominent role in the clearance of many viruses and foreign molecules. Large viral particles exit the circulation and enter the liver and splenic sinusoids, where a high density of professional phagocytes is present (24, 27) in the mononuclear phagocyte system, also known as the reticuloendothelial system. Phages may also exert pharmacodynamic effects by influencing the inflammatory properties of mononuclear cells (23, 28, 29). However, neutrophils are also abundant in the liver, spleen, and peripheral tissues and are the most common phagocyte in circulation. Neutrophils have been reported to engulf viruses (30, 31). However, the contributions of neutrophils to phage clearance remains unclear.

In this study, we evaluated the PK of i.v. administered *P*. *aeruginosa* phage LPS-5 in an immunocompetent and neutropenic mouse model.

## Results

### Calibration studies to evaluate our ability to assess phage concentrations.

**Figure 1:**
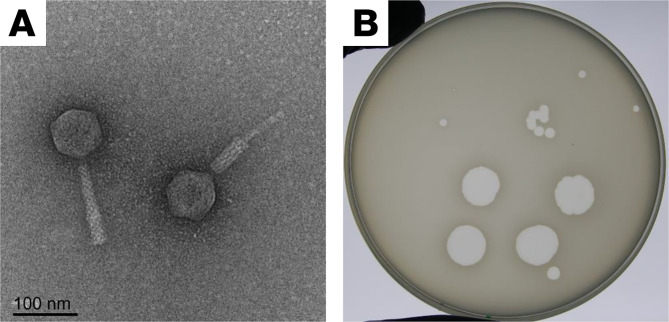
LPS-5 is a tailed phage used in the treatment of drug-resistant infections. (**A**) A representative transmission electron microscopy image of LPS-5 (original magnification, ×80,000) is shown in extended and contracted states. (**B**) Lytic plaque morphology for LPS-5 on *Pseudomonas aeruginosa* strain PAO1.

We chose to study phage LPS-5, an antipseudomonal phage recently included in a human clinical trial of phage therapy, the CYstic Fibrosis bacterioPHage Study at Yale (CYPHY; ClinicalTrials.gov NCT04684641). LPS-5 is a member of the *Pakpunavirus* family and uses Pseudomonal LPS for viral entry (14). A representative transmission electron micrograph of LPS-5 and an image demonstrating LPS-5 plaque morphology on a lawn of *P*. *aeruginosa* PAO1 are shown in Figure 1.

**Figure 2:**
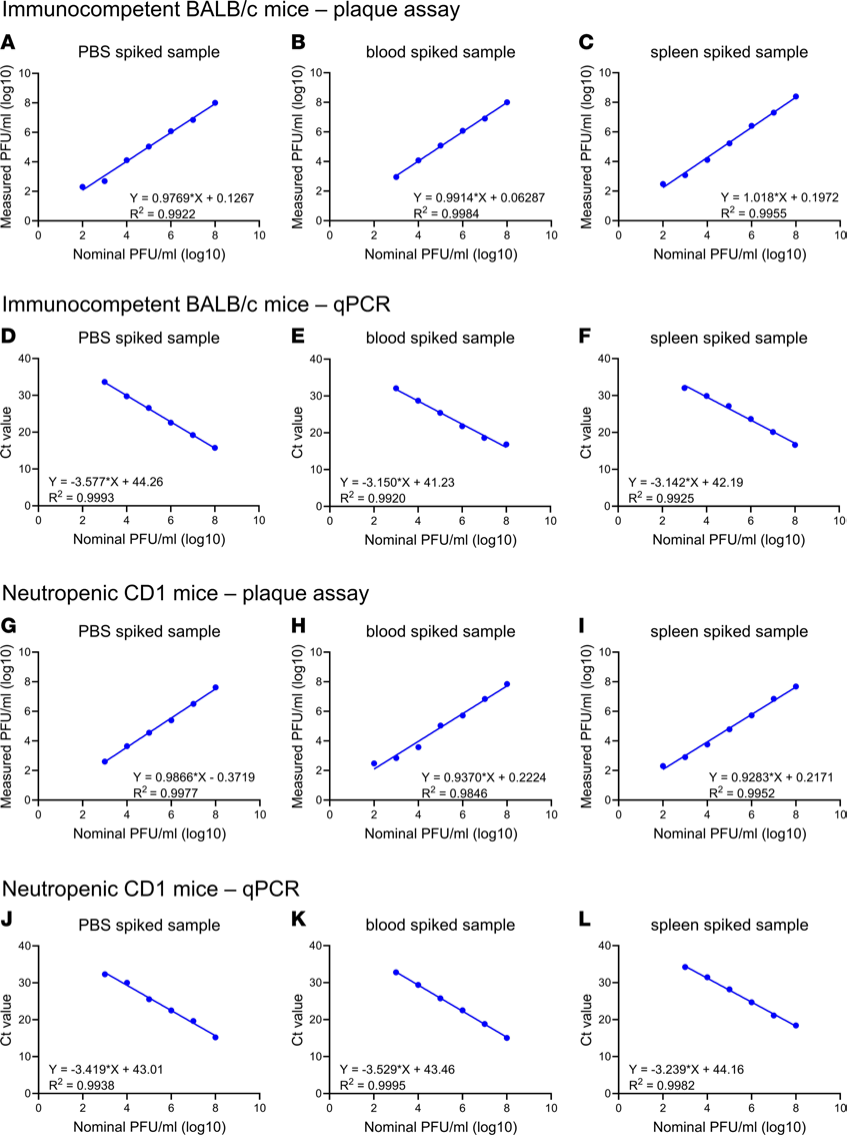
Calibration curves for phage titrations in blood and spleen. To assess our ability to accurately quantify phages in blood and tissue samples, we performed titration studies using plaque assays and qPCR assays as our readouts. For these studies phages with established, nominal concentrations were added to PBS, blood, or spleen tissue collected from (**A**–**F**) immunocompetent BALB/c mice or (**G**–**L**) neutropenic ICR mice. These were then analyzed using (**A**–**C** and **G**–**I**) plaque assays or (**D**–**F** and **J**–**L**) qPCR. Log-transformed phage titers and Ct values are plotted over log-transformed normal phage titers. A linear regression was performed.

We first sought to assess the linearity, accuracy, and precision of plaque assays and quantitative PCR (qPCR) to measure phages in sera and tissues collected from immunocompetent BALB/c and neutropenic CD1 mice over a broad range of phage concentrations. A known amount of LPS-5 (10^3^, 10^4^, 10^5^, 10^6^, 10^7^, 10^8^ PFU/sample) was added to PBS (saline), whole peripheral blood, or excised spleen tissue. Spleen tissues were homogenized after adding phages. These were then analyzed by plaque assays (Figure 2, A–C and G–I) or qPCR (Figure 2, D–F and J–L).

For the qPCR studies, the median accuracy in BALB/c samples was determined to be 101% (range, 90%–115%) for spleen and 93% (range, 88%–94%) for blood, and the uncertainty was determined to be 6.4% for spleen and 0.7% for blood. The median accuracy in CD1 samples was determined to be 88% (range, 85%–91%) for spleen and 100% (range, 100%–102%) for blood, and the uncertainty was determined to be 2.7% for spleen and 0.8% for blood. The limit of detection was determined to be 200 PFU/sample. The lower limit of qualification (LLOQ) was determined to be 1,000 PFU/sample. These data are strong indications of excellent linearity and reproducibility using these approaches.

### The PK of phage LPS-5 in immunocompetent BALB/c mice.

Having established methods to assess phage levels in mouse tissues, we next sought to determine the PK of phage LPS-5 in immunocompetent mice. For these studies, we used BALB/c mice, a well-established mouse strain commonly used in pharmacology studies.

**Figure 3:**
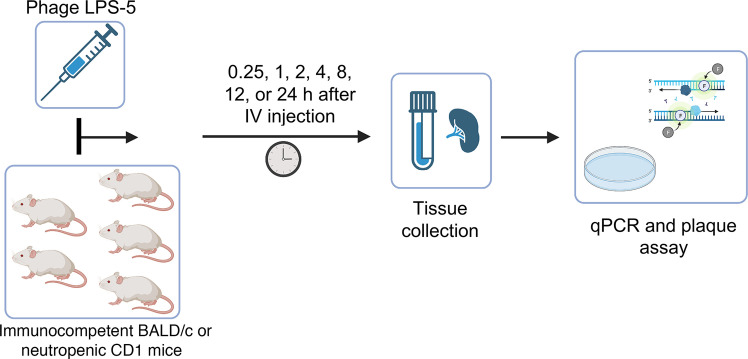
Schematic of biodistribution and pharmacokinetics experiment. In these studies, phage LPS-5 was administered i.v. as a single dose (0.1 mL/mouse at 1.0 × 10^11^ PFU/mL) to immunocompetent BALB/c mice or neutropenic CD1 mice. Animals were sacrificed after 0.25 hours, 1 hour, 2 hours, 4 hours, 8 hours, 12 hours, or 24 hours. At every sampling time point, 5 mice were sacrificed, and the phage concentration was measured in whole blood and spleen homogenates by qPCR and plaque assay. Noncompartmental analysis was conducted to estimate pharmacokinetic parameters.

**Figure 4:**
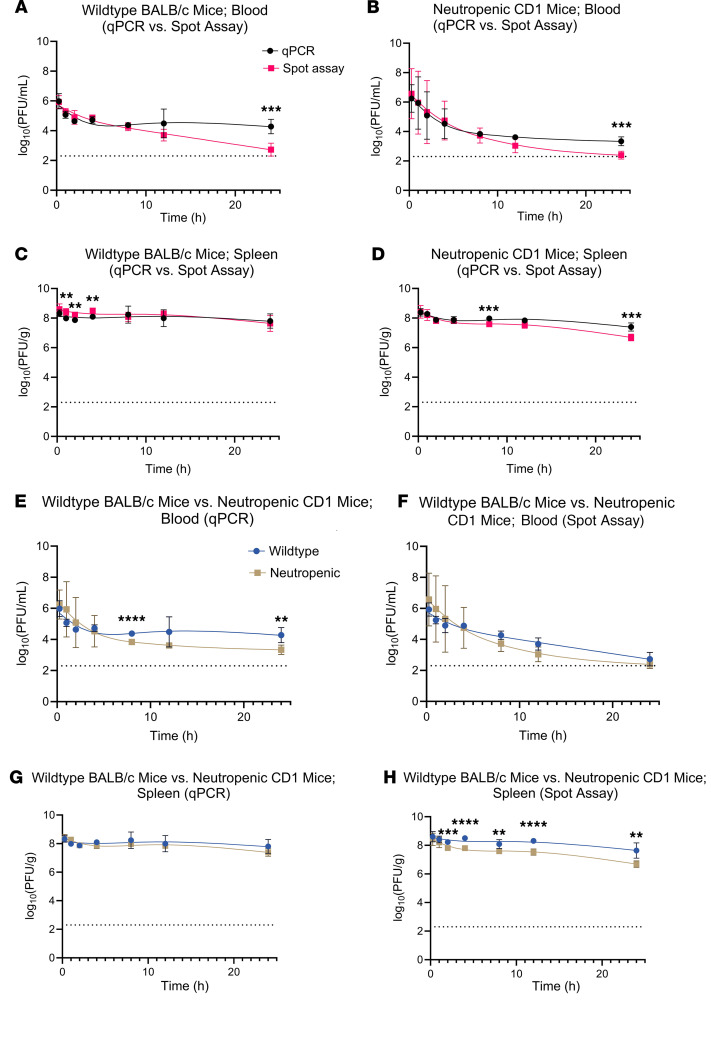
Pharmacokinetics and biodistribution of LPS-5 bacteriophage in blood and spleen tissue over time in BALB/c (immunocompetent) and CD1 (neutropenic) mice. Mice received 0.1 mL of 1.0 × 10^11^ PFU/mL LPS-5 phage suspension i.v. by tail vein injection. Five mice were sacrificed at 0.25, 1, 2, 4, 8, 12, and 24 hours for measurement by qPCR and plaque assays of blood and spleen tissue homogenates. 35 immunocompetent mice and 35 neutropenic mice were used. Geometric mean ± SD is plotted over time. (**A**–**D**) Blood pharmacokinetics (**A** and **B**) and spleen biodistribution (**C** and **D**) of LPS-5 in immunocompetent BALB/c versus neutropenic CD1 mice, as determined by qPCR (**A** and **C**) or spot assay (**B** and **D**). (**E**–**H**) Comparison of PK curves derived from qPCR and spot assay in blood (**E** and **F**) and spleen (**G** and **H**) in immunocompetent BALB/c (**E** and **G**) and neutropenic CD1 mice (**F** and **H**). The dotted lines indicate lower limit of detection (LLOD). **P < 0.001, ***P < 0.0001, ****P < 0.00001, *t* tests adjusted for multiple comparisons using Holm-Šídák method. Statistical analyses were performed on means of log-transformed values due to right-skewness of PK data.

Using these animals, we examined phage PK using the approach shown in Figure 3. LPS-5 phage was administered by i.v. tail vein injection at a single dose (0.1 mL/mouse at a concentration of 1.0 × 10^11^ PFU/mL) to BALB/c mice. Animals were sacrificed after 0.25 hours, 1 hour, 2 hours, 4 hours, 8 hours, 12 hours, and 24 hours. Phage concentrations were measured in whole blood and spleen homogenates (Figure 4, B and D) using plaque assays and qPCR. The concentration over time was graphed and noncompartmental analysis was performed.

The concentration-time profile, as measured by plaque assay of active LPS-5 after i.v. administration in conventional BALB/c mice, is shown in Figure 4A. The C_max_ of active phages in blood in BALB/c mice was 8.55 × 10^5^ PFU/mL, approximately 0.01% of the injected dose, suggesting extremely rapid clearance (Figure 4B) of phages from the bloodstream. Circulating active phages demonstrated first-order elimination with a terminal *t*_1/2_ of 3.24 hours. By 24 hours, very low concentrations of active phage remained in the blood (C_24_ = 539 PFU/mL). High concentrations of active phages were rapidly achieved in spleen tissue (C_max_ = 2.44 × 10^8^ PFU/g) and remained relatively high over the 24-hour period of measurement (C_24_ = 4.91 × 10^6^ PFU/g) (Figure 4C). Other pharmacokinetic parameters are listed in Table 1.

Together, these data suggest that bacteriophages leave the circulation rapidly after administration and accumulate in filtration organs, including the spleen. These data suggest that the phage is removed from the bloodstream by the mononuclear phagocyte system.

### The PK of phage LPS-5 in neutrophil-deficient mice.

We then asked how neutropenia affected phage PK and uptake by the spleen. For these studies, we used the well-established CD1 neutropenic mouse model (32, 33).

Here again, LPS-5 phage was administered by i.v. tail vein injection at a single dose (0.1 mL/mouse with a concentration of 1.0 × 10^11^ PFU/mL) to CD1 mice. Animals were then sacrificed at various time points, and phage concentrations were measured in whole blood and spleen homogenates.

In general, we observed a highly similar PK profile in CD1 mice compared with BALB/c mice (Figure 4, E–H). The C_max_ of phages in blood in CD1 mice was 3.75 × 10^6^ PFU/mL, approximately 0.05% of the injected dose, again suggesting extremely rapid clearance. C_24_ was low, at 249 PFU/mL. As with the BALB/c mice, we once again observed first-order elimination of active phage particles in blood from CD1 mice, with a *t*_1/2_ in the blood of 3.94 hours (Figure 4C). The concentration of active viral particles decreased in spleen tissue by approximately 1 log over the measured duration, with a C_max_ in the spleens of 4.05 × 10^8^ PFU/g and a C_24_ of 4.33 × 10^7^ PFU/g (Figure 4D). Other pharmacokinetic parameters are listed in Table 1.

As with the BALB/c mice, these data indicate that in neutropenic CD1 mice, LPS-5 bacteriophages leave the circulation rapidly after administration and accumulate in other compartments like the spleen.

In comparing the values for neutropenic CD1 mice to those for conventional BALB/c mice, we note that the values for C_max_, C_24_, and *t*_1/2_ are similar among these strains (Figure 4, E–H). For example, the *t*_1/2_ of phages in blood in BALB/c and CD1 mice is 3.24 hours and 3.94 hours, respectively. There was a relatively modest 3- to 4-fold increase in the AUC from the time of dosing extrapolated to infinity (AUC_inf_) in the blood and spleen tissue of neutropenic mice, but the overall PK are similar between the two groups of animals. These data suggest that neutrophil-mediated phagocytosis is not the major determinant of phage clearance.

### Phages are inactivated in circulation.

Our data reveal a substantial discrepancy in phage quantity over time when measured by qPCR versus plaque assay in blood, especially at later time points. The C_24_ in the blood of the BALB/c mice measured by qPCR was 1.89 × 10^4^ PFU/mL versus 539 PFU/mL measured by plaque assay. This is a difference of around 35-fold. In the CD1 mice, the C_24_ was 2,160 PFU/mL measured by qPCR versus 249 measured by plaque assay. This is equivalent to a 9-fold difference, approximately (Table 1).

These data indicate that factors in circulation reduce the number of functional phages (measured by plaque assay) compared with the number of functional phages plus phage fragments (measured by qPCR). Phages stay active for longer in the spleen than in the blood. The AUC_24_ in the spleen of the BALB/c mice measured by plaque assay was 1.01 × 10^9^ PFU/g versus 1.05 × 10^6^ PFU/mL in the blood. A similar observation was made in neutropenic CD1 mice (Table 1). The fact that the phages stay active for longer in the spleen than in the blood suggests that soluble factors in the blood inactivate phage particles. Additionally, we can conclude that phages can retain activity in spleen tissue over the measured time, whereas their activity drops in the blood compartment.

## Discussion

Here, we have examined the role of neutrophils in bacteriophage PK using well-established mouse models and LPS-5, a phage belonging to Pakpunavirus family that has been used in a recent human clinical trial (34). We observe that the PK of phages in the blood and spleen is only minimally affected by neutropenia. These data suggest that neutrophil endocytosis is unlikely to be a major cause of phage clearance within blood. Indeed, the small size of phage particles (25–250 nm) may be too small to trigger conventional phagocytosis mechanisms, as this typically involves particles of 2–3 μm (35, 36) or engagement of specific receptors in receptor-mediated endocytosis.

Our data reveal reduced recovery of plaque-forming phages from serum, relative to qPCR. At 12 hours and 24 hours, the concentration by qPCR was approximately 3- to 9-fold and approximately 9- to 35-fold higher than that found using plaque assay, respectively. This suggests that substantial functional inactivation of circulating phages occurs over time, perhaps due to circulating or tissue-specific immune factors. To this point, there are reports indicating that bacteriophages are susceptible to complement-mediated inactivation via direct destruction or opsonization (37, 38). Since these mice had not seen LPS-5 phage before, it is extremely unlikely that they possessed neutralizing antibodies against these phages. Alternatively, phage degradation over time will lead to loss of plaque formation. As qPCR is unable to distinguish intact and degraded phages, this would further contribute to the discrepancy seen between plaque formation and qPCR detection.

These studies have several implications for phage therapy. First, these data suggest that clearance of active phage particles from peripheral blood is fairly rapid. Second, they suggest that approaches to identify and nullify factors involved in phage clearance in circulation might further improve phage PK. Third, these studies reveal that neutrophils do not contribute to the systemic clearance and in vivo PK of LPS-5 phage.

There are several limitations to acknowledge. First, this study focused on the characterization of phage PK in the absence of bacterial infection. This allowed us to assess phage clearance by the host independent of phage replication. However, the inflammatory response following bacterial infection could dramatically impact the biodistribution and PK of phages. Moreover, these studies did not evaluate the impact of phage replication within their host bacteria on phage biodistribution and PK. Second, effects other than neutropenia may have been induced by cyclophosphamide treatment. These issues and the impact of phage replication on PK were addressed in a recent review (39). Third, due to extremely rapid clearance of phages from the bloodstream, these studies suggest the utility of measuring very early time points (1–15 minutes after administration). Future studies will calculate the PK of phages in other tissue compartments beyond the spleen and blood, include early time points, and will extend this work to other phages and more complex dosing regimens. Beyond these limitations, additional questions remain. These studies were done with i.v. administered phages. The kinetics and biodistribution of phage following oral or nebulized administration, which are common alternative used routes of phage treatment, remain to be determined. How the findings with LPS-5 reported here compared with other phages, including those within the same family, is also unknown. Additional studies will need to assess how generalizable distribution patterns and kinetics are among phages, particularly those within the same family.

## Methods

### Sex as a biological variable

Sex was not considered a biological variable in murine studies. For in vivo studies, only female mice were used due to practical considerations.

### Chemicals and reagents

The following materials were used in these studies: 0.9% NaCl (Sin-Tong), 2× Power SYBR Green PCR Master, Bacto Agar (214040, BD), disodium phosphate (Na_2_HPO_4_, 71640, MilliporeSigma), LB Broth (Lennox) (240230, BD), magnesium sulfate (MgSO_4_, M2643, MilliporeSigma), nutrient agar plates (CMP0101312, CMP), nutrient broth (DIFCO), PBS tablet, pH 7.4 (P4417, MilliporeSigma), potassium phosphate monobasic (KH_2_PO_4_, P5379, MilliporeSigma), sodium chloride (NaCl, S7653, MilliporeSigma), TANBead nucleic acid extraction kit (M665A46, OptiPure Viral Auto plate), tryptic soy broth (211825, BD), and water for injection (Tai-Yu).

### Phage suspension

Phages were propagated using techniques that are well established at Felix Biotechnology. Briefly, we infected planktonic cultures of bacteria with phage until clearing was observed relative to a noninfected control culture, removed gross bacterial debris by centrifugation (8,000*g*, 20 min, 4°C), and filtered the supernatant through a 0.22 μm PES membrane (Corning, product 4311188). The supernatant was treated with 5 U/mL Benzonase nuclease (MilliporeSigma, E8263) overnight at 37°C to digest free DNA. Phage was precipitated by the addition of 0.5 M NaCl + 4% w/v polyethylene glycol 8000 (MilliporeSigma, PHR2894) overnight at 4°C. Precipitated phage was then pelleted by centrifugation (14,000*g*, 20 min, 4°C), washed in 30 mL Tris-EDTA buffer (10 mM Tris-HCl, 1 mM EDTA, pH 8.0), and repelleted by centrifugation (14,000*g*, 20 min, 4°C). The phage pellet was then resuspended in 3 mL of the buffer appropriate to that phage/experiment and dialyzed against 4 L of the same buffer 3 times through a 10 kDa dialysis membrane to remove residual salts and PEG. Felix Biotechnology supplied the LPS-5 bacteriophage stock solution at 1.0 × 10^11^ PFU per mL and the heat-inactivated phage solution. Heat inactivation was achieved by treating phages at 70°C for 30 minutes. No plaque formation was observed following heat inactivation.

Pharmacology Discovery Services (PDS) then stored the phages at 4°C. Dosing solutions for injection were prepared by diluting the stock solution in filtered phage buffer (144 mg/L KH_2_PO_4_, 421.62 mg/L Na_2_HPO_4_, 9,000 mg/L NaCl, 1,203.6 mg/L MgSO_4_) within 1 hour before administration on each dosing day. The phage dose solutions were quantitated twice by plaque assays.

### Animals

Specific pathogen–free immunocompetent female BALB/c mice, 7–8 weeks of age, were used for the PK study. Mice were housed for 3 days in quarantine prior to the PK study. For neutropenic studies, specific pathogen–free female Institute of Cancer Research Hsd:ICR (CD1) mice, weighing 22 ± 2 g, were used. Animals were immunosuppressed by 2 intraperitoneal injections of cyclophosphamide, the first at 150 mg/kg 4 days before infection (day –4) and the second at 100 mg/kg 1 day before infection (day –1), prior to infection on day 0 per the industry-standard methods (40). PDS determined that this cyclophosphamide treatment schedule resulted in neutropenia (<100 neutrophils/μL) until day 2 after injection. All mice were sourced from BioLASCO Taiwan, an Association for Assessment and Accreditation of Laboratory Animal Care–certified Charles River Licensee and rodent breeder.

LPS-5 phage was administered i.v. via tail vein injection at a single dose (0.1 mL/mouse with a concentration of 1.0 × 10^11^ PFU/mL) to BALB/c mice or CD1 mice. Animals were observed at 5–15 minutes after i.v. dosing to detect acute toxicity, which would have been recorded. No toxicity was observed. Animals were then sacrificed after 0.25 hours, 1 hour, 2 hours, 4 hours, 8 hours, 12 hours, or 24 hours. For every sampling time point, 5 mice were sacrificed. Phage concentration was measured in whole blood and spleen homogenates using a plaque assay and qPCR.

### Blood and tissue collection

Animals were euthanized via CO_2_ at the time points noted for blood and tissue collection. Blood samples were collected by cardiac puncture under CO_2_ euthanasia using lithium heparin blood collection tubes. The blood samples were stored on ice and divided into 2 vials, one for PFU enumeration by plaque assays and one for qPCR.

Tissues were aseptically recovered, blotted dry, weighed, and then combined with 1 mL PBS followed by homogenization with a Polytron homogenizer (10,000 × rpm for 15–30 seconds). The homogenized samples were centrifuged, and the supernatant was transferred to 2 vials, one for PFU enumeration by plaque assays and one for qPCR. All samples were stored on ice during processing which was completed within 1 hour.

### Plaque assays

Plaque assays were used to quantify the number of infectious phage particles per mL. Serial 10-fold dilutions of plasma and clarified homogenates were prepared in the phage dilution buffer. The assay was conducted on rectangular Onewell bottom agar plates (128 × 86 mm), containing LB agar (1.5%) with 10 mM MgSO_4_. 10 mL of top agar was prepared for each plate by combining 300 μL of overnight culture of *P*. *aeruginosa* (PAO1) with 10 mL molten top agar medium (LB broth medium with 10 mM MgSO_4_ and 0.75% agar). The overnight culture of *P*. *aeruginosa* strain PAO1 was grown in LB with 10 mM MgSO_4_. Seeded top agar, approximately 10 mL, was spread evenly over the upper surface of the bottom agar plate. After solidification, 5 μL of each sample dilution was pipetted onto the surface of the top agar in duplicate and then left to dry. The plate was incubated at 37°C for 18 hours and then plaques were counted on the spots with 1–50 distinguishable plaques to calculate PFU values. The number of plaques per dilution was tabulated and the PFU/g tissue or PFU/mL blood was calculated and reported.

### Transmission electron microscopy

Transmission electron microscopy (TEM) imaging was done as previously reported (41). In brief, the size and morphology of phages were examined with TEM using a JEOL JEM1400 (JEOL USA Inc.) at 80 kV. 5 μL of diluted phage solution was dropped onto carbon-coated copper grids (FCF-200-Cu, Electron Microscopy Sciences). After 3 minutes, the grid was dipped into a ddH_2_O droplet, and then 1% uranyl acetate was added to the sample and allowed to dry for 15 minutes before performing microscopy.

### qPCR

#### Sample preparation.

Nucleic acid was extracted from the collected blood samples and clarified tissue homogenates using the TANBead Nucleic Acid Extraction kit with the Maelstrom 4810 fully automated DNA/RNA extraction system, as per the manufacturer’s instructions

#### qPCR.

qPCR was conducted with 2× Power SYBR Green PCR Master Mix (Thermo Fisher) using the standard assay components of the kit. The qPCR reactions were performed on a ViiA 7 Real-Time PCR System (Applied Biosystems) using a 96-well format. Assay wells were sealed with optical films and then briefly centrifuged to consolidate the droplets from the sides of wells. Thermal cycling included an initial 10-minute heat activation of the polymerase, followed by 40 repeated cycles of DNA denaturation, primer annealing, and target elongation. The MCP_set2 primer set supplied by Felix Biotechnology was used for qPCR. The MCP_set2 primer set targets nucleotides 5,223–5,367 encoding the major capsid protein of the phage and yields a 145 bp product with a melting temperature of 81.51°C (MCP_set2_F: CGCAACTGGGCTAACACCGC and R: GGTTGGTCAGGTCGAAGCC).

#### Condition optimization.

qPCR condition optimization by analysis of melting temperatures was conducted to inspect for the specificity of the amplified product. To assess specificity, qPCR was performed with primer pairs for each phage using nucleic acid prepared from infected *P*. *aeruginosa* mouse blood. The qPCR signal and the threshold cycle value (Ct) was measured in triplicate in each of these samples, and the signal-to-background values were determined. Tests were conducted to confirm that the primers would only yield amplicons from template samples that contained phage DNA. Template samples containing mouse blood or bacteria, but lacking phage, were tested with the expectation that they would not yield an amplification signal. A dilution series of the phage were tested to define the linear range of the qPCR assay for each phage, the lower limit of detection and LLOQ, the upper limit of quantification (if measurable), and the efficiency of amplification. The experiment assessed the linearity of the qPCR signal from the phage over 10 concentrations: 0, 10, 25, 50, 10^2^, 10^3^, 10^4^, 10^5^, 10^6^, 10^7^, and 10^8^ PFU. Blood samples and homogenized tissue samples were spiked with phage dilutions at these 10 concentrations to mimic samples from treated animals. PBS, blood, and homogenized spleen without spiked phage were included as negative controls. DNA was extracted and measured as described above. The qPCR analysis of each sample was conducted in triplicate to assess intra-assay variability.

### qPCR standard curve

Calibration curve samples were generated as described above by spiking homogenized tissues or blood samples with phage dilutions of defined concentrations in an 8-point dilution series, ranging from 10^1^ to 10^8^ PFU/mL. Triplicate qPCR measurements of each phage were conducted for each sample. For calibration curve samples, the Ct values were graphed as a function of the nominal phage concentration (data not shown). The concentration of each phage in the tissue sample of unknown concentration was determined by comparing the measured Ct values against the calibration curve from the same biological matrix, per the formula below.

Each qPCR condition included 4 QC samples that were prepared according to the same extraction procedure and the same test occasion as the test samples. Clarified tissue or blood homogenates were spiked at 4 concentrations: the LLOQ and the low, mid, and high concentrations of phage over three replicate samples. Controls included no phage vehicle samples. The no-phage blanks were evaluated for the presence of signal artifacts due to contamination.

The Ct values for the dilution samples were graphed as a function of the logarithm of the nominal phage concentration (PFU/mL). The amplification efficiency, E, was determined from the slope per the equation below. Regression analysis was conducted to determine the r values to assess linearity. Concentrations of phage (PFU/mL) in each sample were back-calculated from the calibration standard curve, per the equation below. The calculated concentrations were compared with the nominal concentrations to calculate the assay precision using the following equations: efficiency = 10^–1/slope^ – 1; DNA phage equivalence (PFU/mL or PFU/tissue) = 10^(Ct^ ^value^^–^^Y^ ^intercept)/slope^; accuracy = measured/nominal × 100%; and precision = SD/mean × 100%.

#### Acceptance criteria.

The dilution series yielded an *r* value of 0.98 or higher over 7 or more concentrations. The precision was 85%–115% for 70% of the calibration curve samples. The amplification efficiency was 85%–105%. The linear range should be between 10^3^ and 10^8^ PFU/mL or PFU/tissue of samples.

The concentration of each phage titer was determined in the blood and tissue samples. The concentration-time profile of phage measured with qPCR was characterized as described above for the plaque assay. The outcomes of the qPCR and the phage titration assays were compared.

### PK data analysis

For each time point, the mean of log-transformed phage concentrations from 5 mice was calculated. Statistical analyses were performed on the mean of log-transformed values due to right-skewness of the data. The antilog of the mean concentration was then taken and used for subsequent noncompartmental analysis. Plots of phage concentration in blood and spleen tissues over time were generated with GraphPad Prism software. C_max_ and C_24_ were taken directly from the data. The apparent terminal elimination rate constant (λ_z_) was determined by linear regression analysis of the terminal portion of the log plasma concentration-time curve. The terminal half-life (*t*_1/2_) was calculated as ln(2)/λ_z_. AUC_last_ was found using the linear trapezoidal method. Summation of AUC_last_ plus the concentration at the last measured point divided by λ_z_ yielded AUC_inf_. Volume of distribution based on the terminal phase (V_z_) was calculated as dose/(λ_z_ × AUC_inf_) (42).

### Statistics

Graphpad PRISM (version 9) was used to generate graphs and perform statistical analysis. Two-tailed *t* tests were adjusted for multiple comparisons using Holm-Šídák method. Statistical analyses were performed on means of log-transformed values due to right-skewness of PK data. *P* values of less than 0.05 were considered significant.

### Study approval

The animal studies were reviewed and approved through the PDS IACUC (approval PK001-08242021, September 11, 2021). PDS complies with the International Guiding Principles for Biomedical Research and with the European animal welfare standards. PDS facility and PDS procedures have been fully accredited by the Association for Assessment and Accreditation of Laboratory Animal Care International in 2014, 2017, and 2020 (no. 001553). PDS has a current foreign assurance from the Office of Laboratory Animal Welfare (F16-00213, legacy A5890-01), issued by the PHS NIH for vertebrate animal studies. All animal technicians were trained and evaluated for compliance with animal welfare regulations and were required to report violations if they were observed.

### Data availability

Data are available upon reasonable request, subject to institutional review and approval, of the corresponding authors. Values for all data points in graphs are reported in the Supporting Data Values file.

## Author contributions

Conceptualization was provided by RM, PLB, LM, AE, and TD. Methodology was provided by RM, LM, AE, TD, and ARF. Investigation and data processing were provided by LM, JHC, KYL, CCS, YLL, YCY, WTL, AE, ARF, TD, AK, KD, DFA, and PMB. Data analysis was provided by TD, AE, ARF, AK, PLB, and FGB. Writing was performed by PLB, TD, AE, AK, and PMB. AE and TD equally contributed to experimental design, investigation, analysis, and writing; however, AE served as lead first and coordinated all aspects of this submission. AK contributions were less than those of AE and TD.

## Supplementary Material

Supporting data values

## Figures and Tables

**Table 1:**
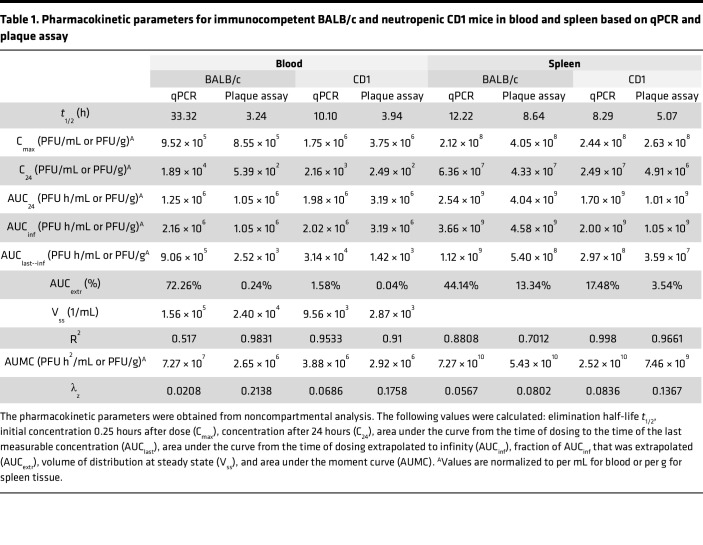
Pharmacokinetic parameters for immunocompetent BALB/c and neutropenic CD1 mice in blood and spleen based on qPCR and plaque assay

## Version 1 10/22/2024

Electronic publication
