# Corrigendum to The contribution of neutrophils to bacteriophage clearance and pharmacokinetics in vivo

**DOI:** 10.1172/jci.insight.213403

**Published:** 2026-10-22

**Authors:** Arne Echterhof, Tejas Dharmaraj, Arya Khosravi, Robert McBride, Lynn Miesel, Ju-Hsin Chia, Patrick M. Blankenberg, Kun-Yuan Lin, Chien-Chang Shen, Yu-Ling Lee, Yu-Chuan Yeh, Wei Ting Liao, Francis G. Blankenberg, Krystyna Dąbrowska, Derek F. Amanatullah, Adam R. Frymoyer, Paul L. Bollyky

Original citation: *JCI Insight*. 2024;9(20):e181309. https://doi.org/10.1172/jci.insight.181309

Citation for this corrigendum: *JCI Insight*. 2026;11(20):e213403. https://doi.org/10.1172/jci.insight.213403

The authorship list has been updated to include Hemaa Selvakumar, Joel Berry, and Max Hopkins. In addition, the Author contribution section has been updated to include the contributions of these authors.

The authors regret the error.

## Version 1 10/22/2026

Electronic publication

